# Feasibility and acceptability of a preoperative exercise program for patients undergoing major cancer surgery: results from a pilot randomized controlled trial

**DOI:** 10.1186/s40814-021-00765-8

**Published:** 2021-01-13

**Authors:** Daniel Steffens, Jane Young, Paula R. Beckenkamp, James Ratcliffe, Freya Rubie, Nabila Ansari, Neil Pillinger, Cherry Koh, Phillip A. Munoz, Michael Solomon

**Affiliations:** 1 Surgical Outcomes Research Centre (SOuRCe), Royal Prince Alfred Hospital (RPAH), Missenden Road, Camperdown, New South Wales 2050 Australia; 2 Faculty of Medicine and Health, Central Clinical School, The University of Sydney, Building 89, Leval 9, Missenden Road, Camperdown, New South Wales 2006 Australia; 3 Institiute of Academic Surgery (IAS), Royal Prince Alfred Hospital, 145-147 Missenden Road, Camperdown, New South Wales 2050 Australia; 4 Faculty of Medicine and Health, School of Health Sciences, The University of Sydney, Lidcombe, New South Wales 2141 Australia; 5 Department of Physiotherapy, Royal Prince Alfred Hospital, Camperdown, New South Wales 2006 Australia; 6 Department of Anaesthetics, Royal Prince Alfred Hospital, Camperdown, New South Wales 2006 Australia; 7 Department of Respiratory and Sleep Medicine, Royal Prince Alfred Hospital, Camperdown, New South Wales 2006 Australia

**Keywords:** Preoperative exercise, Pilot randomized controlled trial, Cancer, Surgery, Feasibility, Acceptability

## Abstract

**Objective:**

To establish the feasibility and acceptability of a preoperative exercise program, and to obtain pilot data on the likely difference in key surgical outcomes to inform the sample size calculation for a full-scale trial.

**Design:**

Pilot randomized controlled trial.

**Setting:**

Royal Prince Alfred Hospital, Sydney, Australia.

**Subjects:**

We included patients undergoing elective pelvic exenteration or cytoreductive surgery aged 18 to 80 years, who presented to the participating gastrointestinal surgeon at least 2 weeks prior to surgery. Patients presenting cognitive impairment, co-morbidity preventing participation in exercise, inadequate English language, currently participating in an exercise program or unable to attend the exercise program sessions were excluded.

**Methods:**

Participants were randomized to a 2–6 weeks preoperative, face-to-face, individualised exercise program or to usual care. Feasibility was assessed with consent rates to the study, and for the intervention group, retention and adherence rates to the preoperative exercise program. Acceptability of the exercise program was assessed with a semi-structured questionnaire exploring the advice received and the amount, duration and intensity of the exercise program. In addition, postoperative complication rates (Clavien-Dindo), length of hospital stay and self-reported measures of health-related quality of life (SF-36v2) were collected at baseline, day before surgery and in-hospital up to discharge from hospital.

**Results:**

Of 122 patients screened, 26 (21%) were eligible and 22 (85%) accepted to participate in the trial and were randomized to the intervention (11; 50%) or control group (11; 50%). The median age of the include participants was 63 years. Adherence to the preoperative exercise sessions was 92.7%, with all participants either satisfied (33%) or extremely satisfied (67%) with the overall design of the preoperative exercise program. No significant differences in outcomes were found between groups.

**Conclusions:**

The results of our pilot trial demonstrate that a preoperative exercise program is feasible and acceptable to patients undergoing major abdominal cancer surgery. There is an urgent need for a definite trial investigating the effectiveness of a preoperative exercise program on postoperative outcomes in patients undergoing major abdominal cancer surgery. This could potentially reduce postoperative complication rates, length of hospital stay and subsequently overall health care costs.

**Trial registration:**

ACTRN12617001129370. Registered on August 1, 2017, https://www.anzctr.org.au/Trial/Registration/TrialReview.aspx?id=373396&showOriginal=true&isReview=true

**Supplementary Information:**

The online version contains supplementary material available at 10.1186/s40814-021-00765-8.

## Key messages regarding feasibility

1. What uncertainties existed regarding the feasibility? Its unknown whether a preoperative exercise program is feasible and acceptable for patients undergoing major cancer surgery.
2. What are the key feasibility findings? A preoperative exercise program is feasible and acceptable for patients undergoing major abdominal cancer surgery.
3. What are the implications of the feasibility findings for the design of the main study?

A full-scale randomized controlled trial is warranted to investigate the effectiveness of a preoperative exercise program on postoperative outcomes for patients undergoing major abdominal cancer surgery.

## Introduction

Advanced primary or recurrent gastrointestinal cancers are one of the most deadly cancers worldwide [1, 2]. For selective patients, radical and complex surgical procedures are the only potentially curative treatment options to improve survival. Such surgery includes pelvic exenteration for advanced malignancy within the pelvis which may require surgical removal of the bladder, urethra, colon, rectum, anus and/or sacrum, and Cytoreductive Surgery & Hyperthermic Intraperitoneal Chemotherapy (CRS & HIPEC) which can involve resection of multiple viscera including the small bowel, colon, gallbladder, spleen, uterus and ovaries with the addition of HIPEC to treat peritoneal malignancy [3, 4]. Despite the now acceptable overall postoperative mortality rates, postoperative complications remain a challenge in this patient group. The incidence of postoperative complications following pelvic exenteration and CRS & HIPEC ranges from 40 to 100% [5–7]. Therefore, effective targeted interventions to reduce postoperative complications are needed.

A recent systematic review investigating the association between preoperative physical activity levels and postoperative surgical outcomes has suggested that patients presenting with a higher level of physical activity during the preoperative period experienced improved surgical outcomes [8]. A prospective cohort study investigating the association between preoperative physical activity levels and postoperative complications in patients undergoing pelvic exenteration presented similar outcomes [9]. Patients that reported engagement in walking (odds ratio [OR] 0.96; 95% confidence intervals [CI] 0.92 to 0.99), moderate (OR 0.94; 95%CI 0.88 to 0.99) and vigorous physical activity (OR 0.84; 95%CI 0.71 to 0.98) were less likely to develop postoperative complications [9].

Several randomized controlled trials have investigated the effectiveness of a preoperative exercise intervention on improving surgical outcomes and patient reported outcomes [10]. However, most of these trials focus on patients undergoing surgery for lung cancer. In this population, a preoperative exercise program was effective in reducing postoperative complication by approximately 50% and length of hospital stay by 3 days [10]. There is evidence suggesting that patients undergoing an exercise program before surgery would benefit. However, further investigations are needed for patients undergoing more complex and advanced abdominal and pelvic cancer surgery, such as pelvic exenteration and CRS & HIPEC.

Therefore, the primary aims of this pilot randomized controlled trial was to determine: (a) the feasibility of incorporating a standardised, high intensive exercise program into the pre-operative period for patients undergoing major gastrointestinal surgery; (b) the acceptability of the exercise program to patients; and (c) the acceptability of patients to being randomized to the exercise program or usual care. The secondary aim was to obtain pilot data on the likely difference in key outcomes (post-operative complications, length of hospital stay, post-operative functional capacity and health-related quality of life) to inform the sample size calculation for a definite randomized clinical trial.

## Methods

### Trial design

This study is a single-centre, two arm (1:1 allocation ratio), parallel, pilot randomized controlled trial carried out at the Royal Prince Alfred Hospital, Sydney, Australia. The study protocol was published [11] and registered at ANZCTR (ACTRN12617001129370). This pilot trial was written in accordance to the extension of the Consolidated Standards of Reporting Trials (CONSORT) for randomized pilot and feasibility trials [12, 13]. Ethics approval was obtained from the Sydney Local Health District Human Research Ethics Committee (Protocol No X17–0189 and HREC/17/RPAH282). Informed consent was understood, accepted and signed by all participants included in the trial.

### Participants

Consecutive patients scheduled to undergo pelvic exenteration or CRS & HIPEC at the Royal Prince Alfred Hospital between September 21, 2017, and October 18, 2018, were invited to participate. Eligible patients fulfilled the following criteria: (i) aged 18 to 80 years, (ii) underwent elective pelvic exenteration or CRS & HIPEC, and (iii) presented to the participating gastrointestinal surgeon at least 2 weeks prior to planned surgery. Patients were excluded from the trial if they presented any of the following criterion: (i) cognitive impairment such that they were unable to provide informed consent (assessed by the treating surgeon in addition to history taken from patient’s carer), (ii) co-morbidity preventing participation in exercise (i.e. major cardiac, respiratory or musculoskeletal disease), (iii) inadequate English language precluding the completion of the trial’s outcome measures, (iv) current participation in an active exercise program similar to the proposed intervention and (vi) unable to attend the exercise program sessions (e.g. living in another state).

### Interventions

#### Preoperative exercise program group

The intervention consisted of a comprehensive preoperative exercise program and usual care. The main aim of the preoperative exercise program was to increase aerobic capacity and peripheral muscle endurance, muscle strength and respiratory muscle function and to educate participants to perform the exercises at home. Therefore, the exercise program was delivered using three main components:

(i) *Supervised, individualised, and progressive exercise*: The preoperative exercise program consisted of 1 h individualised (one-to-one) training session with a registered physiotherapist, once a week, for 2 to 6 weeks, delivered at the colorectal ward gym. The individualised exercise program was tailored to each participant and was based on their baseline health assessment, physical activity level, presence of co-morbities and medical history. Each of the training sessions included warm-up (walking/cycling), aerobic and endurance (cycle ergometer, treadmill and rowing machine), respiratory (pursed lip breathing and deep breathing exercises), muscle-strength exercises (squats, push-ups, shoulder press, hamstring curls, dumb-bell deadlift, biceps curl and overhead triceps extension) and cool-down activities (triceps, lower back, hip flexors, quadriceps, hamstrings and calf muscles). The aerobic and endurance exercises were performed at 12 to 14 on the rated perceived exertion scale (Borg scale) [14], and the strength training were performed at an intensity of 40 to 60% of the 1-repetition maximum [15]. Participants were re-assessed at each session, and exercises were progressed or adapted by the study physiotherapist if required.

(ii) *Home exercises*: Home-based functional exercises were prescribed by the study physiotherapist during the face-to-face sessions (60 min, 4 times a week). The main aim of the home exercises were to increase aerobic capacity and respiratory muscle function, including exercises with resistance provided by bodyweight, including push ups, squats and step ups. Instructions sheets were provided by the study physiotherapist to help participants with the home exercises. A diary was maintained by the participant with the number of days they have exercised.

(iii) *Daily physical activity advice*: In addition to the home exercise, participants were given an activity tracker (Fitbit®) and encouraged to walk continuously for at least 30 min daily. Patients were asked to keep a diary with the number of days they have walked for ≥ 30 min. The activity tracker was used only during the preoperative exercise period (e.g. 2 to 6 weeks prior to surgery).

#### Control group

Participants allocated to the control group received usual care which consisted of nutritional counselling and advice on smoking cessation and reduction of alcohol intake. No specific exercise advice was provided, and patients were instructed to maintain their normal daily activities.

### Outcomes

Outcome measures were assessed at baseline, the week before surgery, intra-operatively, 10 days postoperative and at pre-discharge from hospital.

#### Primary outcomes

Feasibility and acceptability of the preoperative exercise program were the main outcomes assessed in this pilot trial. Feasibility was determined by (i) number of eligible participants recruited to the trial, (ii) retention rate (defined as the percentage of participants who completed the trial) and (iii) exercise adherence rates (defined as the percentage of exercise sessions attended by those who were randomized to the intervention group). Adherence to the preoperative exercise program was recorded using attendance records (recorded by the study physiotherapist) and participant exercise diaries (recorded by the participant). This pilot trial would be deemed feasible if ≥ 70% of eligible participants are successfully recruited to the trial, ≥ 90% of the recruited participants complete the trial and ≥ 80% of the exercises sessions are attended by the participants.

Acceptability to the preoperative exercise program was assessed with a semi-structured questionnaire administered at pre-discharge. The semi-structured questionnaire contained 11 questions and participants used a 5 point Likert Scale to express how much they agree or disagree with each statement. In addition, patients were asked how satisfied they were with the preoperative exercise program and if the program negatively affected them. We have also taken the opportunity to seek further feedback about the preoperative exercise program (open-ended question). The questionnaire was developed for this pilot trial, and was pilot tested in 5 patients before the implementation in the trial.

#### Secondary outcomes

Secondary outcomes included the rate of participants developing in-hospital complications (defined as any deviation from the normal postoperative course and classified according to the Clavien-Dindo classification) [16, 17], length of hospital stay, functional capacity (assessed with the 6-min walking test) [18], physical activity (assessed with the International physical Activity Questionnaire – Short Form) [19], quadriceps strength (assessed with the Isometric Quadriceps Strength Assessment and the Five Times Sit to Stand Test) [20] and health-related quality of life (assessed with the Short Form 36 version2) [21]. In addition, adverse events (defined as any undesirable event such as injury, falls, discomfort or pain) was collected throughout the trial period.

#### Other outcomes

Other outcomes assessed included Cardiopulmonary Exercise Test (CPET) [22], Self-Efficacy Scale [23], Depression, Anxiety, Stress Scale (DASS) [24] and pain intensity (Numerical Pain Rating Scale) [25].

### Sample size

The main aim of this pilot trial was not to evaluate the effectiveness of the preoperative exercise program, but to provide information on the feasibility and acceptability of the preoperative exercise program in the targeted population. We estimated that a full-scale trial would require approximately 172 participants. This would provide 90% power to detect a difference of 25% in postoperative complication rates between intervention and control groups. These calculations were based upon the overall complication rate being 50% in the control group, allowing for up to 5% loss to follow-up and a two-side alpha of 0.05. Therefore, we decided to randomize approximately 20 participants into the intervention and control group. This was estimated based on 10% of the number required for the full-scale trial.

### Randomization

A research assistant from the Surgical Outcomes Research Centre (SOuRCe) not involved in the trial revealed the randomized group allocation after each participant was consented and the baseline assessment was completed. We used a computer-based, random-sequence generator, stratified by surgical procedure (i.e. pelvic exenteration and CRS & HIPEC), carried out on a 1:1 ratio. The independent research assistant prepared consecutive numbered, sealed, opaque envelopes containing the group allocation. All the concealed envelopes were stored in an individualized locked cabinet and were opened in sequence to reveal group allocation. The research assistant was independent from the trial and was not involved in the recruitment process, treatment or assessment of outcome measures.

### Blinding

The study research officer and the physiotherapist who conducted the self-reported questionnaires and physical assessments were blinded to group allocation. The surgeon and other clinical staff responsible for the care of the participants were also blinded to group allocation.

Due to the nature of the intervention (i.e. exercise), it was not possible to blind participants to group allocation. However, participants were instructed to not disclose the group allocation to the treating clinicians and research team.

Furthermore, the statistical analysis was performed by a blinded statistician using concealed groups (i.e. group A and group B). The intervention and control group were revealed once all the analyses had been completed.

### Analytical methods

All study data was stored in a secure password protected Research Electronic Data Capture (REDCap) database [26] with all the analyses conducted using IBM SPSS Statistics version 25 (SPSS Inc., Chicago, IL USA).

All categorical variables were presented as percentage, and continuous variables were presented as median and interquartile range (IQR). Baseline characteristics and secondary outcomes of the intervention and control group were compared using the Mann-Whitney *U* test (for continuous variables) and the chi-square (categorical data with expected value for each cell ≥ 5) test or Fisher’s exact test (categorical data with expected value for each cell < 5).

## Results

### Characteristics of the study sample

The baseline characteristics of the study participants is presented on Table 1. The median age of participants was 63 years (IQR 47.5 to 70.5), and slightly more participants were male (54.5%). Of the included participants, 11 (50%) underwent pelvic exenteration and 11 (50%) underwent CRS & HIPEC. Therefore, the total sample size for this study is 22 participants.

**Table 1:** Baseline demogPlease check if tables are captured and presented correctly.raphics of study population

Variables	Control (***n*** = 11)	Intervention (***N*** = 11)
Age (years)	66.0 (46.0 to 70.0)	62.0 (48.0 to 72.0)
Gender		
*Male*	6 (54.5)	6 (54.5)
*Female*	5 (45.5)	5 (45.5)
Weight (kg)	75.0 (62.0 to 105.3)	77.2 (58.9 to 103.5)
Height (m)	167.0 (159.9 to 170.1)	161.5 (153.6 to 174.2)
Preoperative chemo/radiotherapy	3 (27.3)	5 (45.5)
Tumor type		
*Primary rectal*	--	3 (27.3)
*Recurrent rectal*	5 (45.5)	2 (18.2)
*Pseudomyxoma peritoneii*	3 (27.3)	--
*Appendix adenocarcinoma*	3 (27.3)	3 (27.3)
*Colorectal*	--	2 (18.2)
*Other*	--	1 (9.1)
Type of surgery		
*Pelvic exenteration*	5 (45.5)	6 (54.5)
*CRS + HIPEC*	6 (54.5)	5 (45.5)
Pain	1.0 (0.0 to 4.0)	4.0 (2.0 to 6.0)
CPET		
*VO2 Max*	19.9 (14.7 to 23.0)^c^	18.8 (14.0 to 23.3)^e^
*Anaerobic threshold*	12.0 (10.4 to 13.9)^d^	13.6 (9.6 to 16.4)^f^
6MWD	525.0 (459.0 to 585.0)	490.0 (370.0 to 585.0)
Exercise self-efficacy	36.0 (26.0 to 38.0)	28.0 (26.0 to 33.0)
DASS		
*Depression*	4.0 (0.0 to 7.0)	4.0 (2.0 to 7.0)
*Anxiety*	4.0 (0.0 to 7.0)	6.0 (3.0 to 9.0)
*Stress*	6.0 (0.0 to 9.0)	8.0 (4.0 to 12.0)
Physical activity level		
*Low*	2 (18.2)	3 (27.3)
*Moderate*	5 (45.5)	5 (45.5)
*High*	4 (36.4)	3 (27.3)
Physical activity (min/wk)		
*Sitting*	480.0 (340.0 to 660.0)	480.0 (320.0 to 540.0)
*Walking*	420.0 (200.0 to 1680.0)	420.0 (210.0 to 840.0)
*Moderate*	0.0 (0.0 to 0.0)	0.0 (0.0 to 0.0)
*Vigorous*	0.0 (0.0 to 0.0)	0.0 (0.0 to 0.0)
*MET minute/week*	1386.0 (660.0 to 41580)	1386.0 (792.0 to 3252.0)
Sit-to-stand test	9.8 (7.1 to 12.2)^i^	11.4 (7.6 to 13.1)^j^
Quad test	9.2 (5.4 to 19.6)	7.5 (4.0 to 13.9)^k^
Health-related quality of life (SF-36v2)		
Physical Component Score	49.6 (46.4 to 52.5)	44.1 (39.3 to 47.8)
Mental Component Score	44.3 (37.3 to 56.8)	46.0 (27.6 to 50.6)

^a^*N* = 17; ^b^*N* = 16; ^c^*N* = 7; ^d^*N* = 7; ^e^*N* = 10; ^f^*N* = 9;^g^*N* = 21; ^h^*N* = 12;^i^*N* = 6; ^j^*N* = 10; ^k^*N* = 6. No difference between the intervention and control group was found for all variables. Data presented as median (IQR) or *N* (%)

### Feasibility

#### Number of eligible participants

**Fig. 1:**
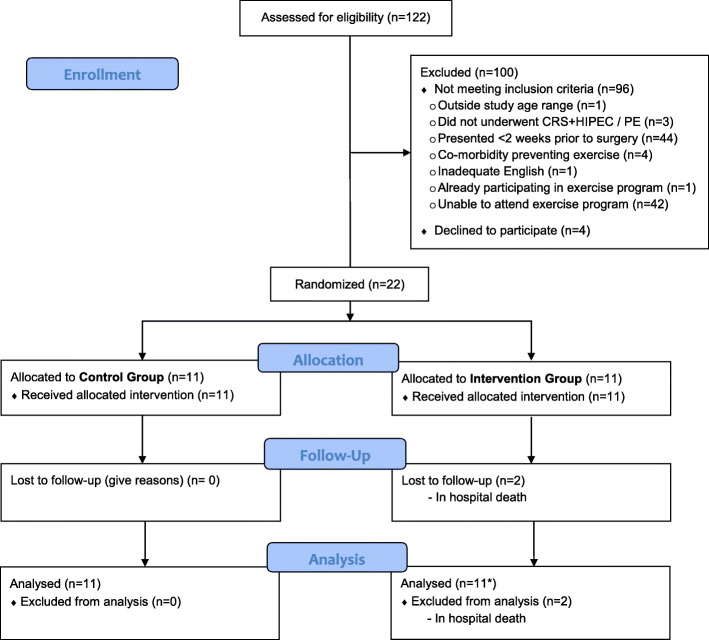
The PEPA Trial CONSORT flow diagram

Between September 21, 2017, and October 18, 2018, 122 patients were identified. Of these, 96 did not meet the inclusion criteria and 4 declined participation. Most patients not meeting the inclusion criteria presented < 2 weeks prior to surgery (44%) or were unable to attend the exercise program due to their residence being too far from our hospital (42%). Therefore, out of the 26 eligible patients, 22 (85%) consented and were randomized to the intervention (*n* = 11) or control group (= 11). The trial CONSORT flow diagram is presented on Fig. 1.

#### Retention

Of the 22 participants who consented and were randomized to the trial, 20 (91%) completed the whole trial. Two participants (9%) died in hospital; therefore, their 10 days postoperative assessment and their pre-discharge assessments were missing. None of the participants withdrew from the trial during the intervention period.

#### Adherence to the preoperative exercise program

Of the 11 participants that were randomized to the intervention group, the mean number of sessions per participant was 4; with participants attending 93% of the total face-to-face sessions (38 out 41). Of the 475 sessions that participants were advised to perform at home (171 home exercises and 304 daily physical activity advices), 302 (64%) were completed.

### Acceptability

The responses of the semi-structured questionnaire are presented in Table 2. Most participants were satisfied with the advice given by the treating physiotherapist (66.7% strongly agree), the amount and intensity of exercise given (66.7% strongly agree) and had no problems in coming to the hospital to perform their weekly face-to-face sessions (44.4% agree and 44.4% strongly agree). Furthermore, most of the participants mentioned that their participation in the preoperative exercise program was influenced by the advice of their surgeon (44.4% agree and 33.3% strongly agree).

**Table 2:** Acceptability of the preoperative exercise program (*n* = 9)

Variables	Strongly disagree	Disagree	Neutral	Agree	Strongly agree
**1.** I was satisfied with the advice received by my physiotherapist / exercise physiologist	--	--	--	3 (33.3)	**6 (66.7)**
**2.** I was satisfied with the amount of exercise received	--	--	--	3 (33.3)	**6 (66.7)**
**3.** My relatives were supporting my participation on the preoperative exercise program	--	--	1 (11.1)	3 (33.3)	**5 (55.6)**
**4.** The recommended home exercises were easy to understand and follow	--	--	1 (11.1)	**4 (44.4)**	**4 (44.4)**
**5.** I had no problems in coming to the hospital to perform my weekly exercises	--	--	3 (33.3)	**4 (44.4)**	2 (22.2)
**6.** The duration of the exercise sessions was just right for me	--	--	--	**7 (77.8)**	2 (22.2)
**7.** The intensity of the exercises was just right for me	--	--	--	**6 (66.7)**	3 (33.3)
**8.** I would recommend the preoperative exercise programme to a friend	--	--	--	3 (33.3)	**6 (66.7)**
**9.** The preoperative exercise programme covered all my needs	--	--	--	4 (44.4)	**5 (55.6)**
**10.** My participation on the preoperative exercise programme was influenced by the advice of my surgeon	--	1 (11.1)	1 (11.1)	**4 (44.4)**	3 (33.3)
**11.** The preoperative exercise programme helped me understand the importance of exercise	--	--	--	4 (44.4)	**5 (55.6)**

Overall, participants were either satisfied (33.3%) or extremely satisfied (66.7%) with the preoperative exercise program, with all providing positive feedback about the pilot intervention: “It helped me manage moving; getting in and out of my hospital bed. I felt stronger and more confident to move after the operation”. None of the participants thought that the preoperative exercise program negatively affected them.

### Secondary outcomes

**Fig. 2:**
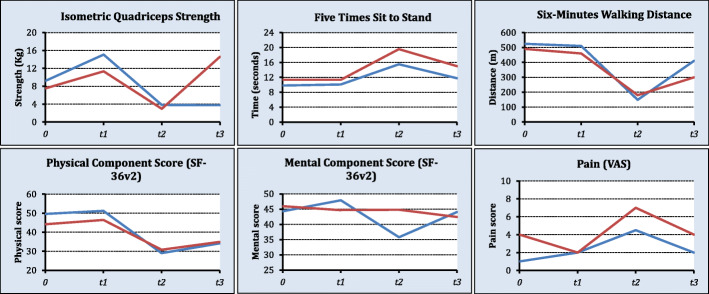
Physical assessment and self-reported outcomes throughout the study period. 0 = baseline, t1 = week before surgery, t2 = 10 days post-operative, t3 = pre-discharge from hospital, red line = intervention group, blue line = control group. Higher scores for quadriceps strength, a 6-min walking distance, physical and mental component scores represent better outcomes. Lower scores for the five times to stand test and pain outcomes represent better outcomes. No statistical difference between groups was reported for all outcomes (*p* > 0.05)

The secondary outcomes are reported in Table 3 and Fig. 2. None of the secondary outcomes presented any statistical difference between the intervention and control group at the pre-discharged period. No adverse events due to the preoperative exercise program were reported throughout the trial period. Estimates for all secondary outcomes can be found on Supplementary Table 1.

**Table 3:** Secondary outcomes (*n* = 22)

Variables	Overall (***n*** = 22)	Control (***n*** = 11)	Intervention (***N*** = 11)
Complication rates	17 (81.0)	7 (70.0)	10 (90.9)
Clavien-Dindo			
*Grade II*	6 (35.3)	2 (28.6)	4 (40.0)
*Grade III*	5 (29.4)	2 (28.6)	3 (30.0)
*Grade IV*	4 (23.5)	3 (42.9)	1 (10.0)
*Grade V*	2 (11.8)	--	2 (20.0)
ICU Stay (days)	6.0 (4.0 to 7.7)^a^	6.0 (3.8 to 9.5)	6.0 (4.0 to 7.5)^b^
Hospital Stay (days)	32.5 (16.2 to 52.5)^a^	29.0 (15.0 to 53.0)	36.0 (15.0 to 52.0)^b^
Functional Capacity (6MWD)	351.0 (240.0 to 431.2)^a^	400.0 (240.0 to 450.0)	300.0 (150.0 to 369.5)^b^
Physical activity Level			
*Low*	14 (73.7)	7 (70.0)	7 (77.8)
*Moderate*	4 (21.1)	3 (30.0)	1 (11.1)
*High*	1 (5.3)	0 (0.0)	1 (11.1)
Physical activity (min/wk)			
*Sitting*	540.0 (60.0 to 1080.0)	870.0 (240.0 to 1245.0)	215.0 (10.0 to 750.0)
*Walking*	70.0 (20.0 to 180.0)	95.0 (0.0 to 187.0)	50.0 (25.0 to 175.0)
*Moderate*	0.0 (0.0 to 0.0)	0.0 (0.0 to 0.0)	0.0 (0.0 to 15.0)
*Vigorous*	0.0 (0.0 to 0.0)	0.0 (0.0 to 0.0)	0.0 (0.0 to 0.0)
*MET minute/week*	231.0 (66.0 to 594.0)	313.5 (0.0 to 619.0)	231.0 (82.5 to 1297.5)
Five times Sit-to-Stand Test	12.6 (9.0 to 16.4)	11.0 (7.2 to 14.4)	15.0 (11.3 to 23.0)
Quadriceps Strength Test	6.3 (3.0 to 28.0)	5.0 (3.0 to 26.0)	14.6 (2.2 to 32.0)
Quality of Life (SF-36v2)			
*Mental Component*	43.6 (35.1 to 9.4)^a^	43.5 (31.1 to 49.3)	42.4 (36.3 to 50.3)^b^
*Physical Component*	34.7 (28.8 to 40.1)^a^	34.6 (32.9 to 44.9)	34.9 (26.1 to 38.7)^b^

Data reported as median (IQR) or *N* (%). *Measured at the pre-discharge day

^a^*N* = 20; ^b^*N* = 9, missing information due to two deaths in hospital. *ICU* intensive care unit, *6MWD* 6-minute walking distance, *SF-36* short form 36 version 2

## Discussion

### Principal findings

To our knowledge, this is the first trial investigating the feasibility and acceptability of a preoperative exercise program in patients undergoing pelvic exenteration and CRS and HIPEC surgery. This pilot trial was shown to be feasible and acceptable by patients undergoing cancer surgery and provides extensive information to be implemented in a full-scale trial. The results of this pilot trial supports the development of a definitive full-scale randomized controlled trial investigating the effectiveness of a preoperative exercise program on improving postoperative outcomes.

### Number of eligible patients recruited

The pilot trial recruitment was completed within 13 months. This was relatively fast, considering the complexity of the trial population. During the recruitment period, most of these patients would have completed a course of chemotherapy and/or radiotherapy, reported high level of pain and fatigue; in addition, at that period, they were recently being informed by their treating surgeon that they would undergo a major procedure or, in some cases, a second procedure (i.e. patients presenting with recurrent cancer), and would have several of their organs, vessels, nerves, ligaments and bone excised [27]. These factors contributed to a challenging recruitment process.

The identification of patients and the recruitment process were enhanced by the engagement of a large number of personnel, including treating surgeons, clinical nurse, allied health team and the research team. Of the 122 patients screened during the 13 months of the study period, 26 patients met the inclusion criteria and were invited to participate; with 22 consenting and randomized to either the preoperative exercise program or usual care. Therefore, of the eligible patients screened, 85% consented and entered the trial. This demonstrates that out of the eligible patients, most were willing to undertake an exercise program before their major surgery. Other comparable pilot trials demonstrated a less favourable result, with some reporting a recruitment rate of 20% and 62% [28, 29].

The most common reasons patients were deemed ineligible were due to presenting < 2 weeks prior to their surgery (46%) or being unable to attend the exercise sessions (44%). Similar results were reported in a randomized controlled trial investigating the feasibility of delivering a preoperative exercise program in patients scheduled for elective colorectal surgery [29]. Of the 198 patients identified, 84 (42%) were ineligible due insufficient time (i.e. presenting < 2 weeks prior to scheduled surgery). To overcome this issue on the full-scale trial, an earlier contact with the patient (e.g. initial consultation with treating surgeon) during the work up period may be required. The average work up period for these patients is around 8 to 6 weeks prior to surgery. Furthermore, to accommodate the trial for patients that were unable to attend the face-to-face sessions at the local hospital, the piloted preoperative exercise protocol should be provided by a local physiotherapist or exercise physiologist. This modification to the intervention, if proven effective, would improve the reach of the program to include patients referred for surgery from across the country.

### Retention to the preoperative exercise program

Most of the patients randomized (91%) remained in the trial until completion. This is in line with previous research, where retention rate was reported at 83% [29]. In the current pilot trial, two participants randomized to the intervention group died in hospital, and therefore, their postoperative outcome assessments were missed. The in-hospital mortality rate for this patient group is usually < 1%, [30] so post-operative mortality will be closely monitored in a future trial. It is also important to note that none of the participants withdrawn from the trial. A challenge in randomized controlled trials is to recruit and retain participants until completion. Some of the issues related to retation rate includes reduction in statistical power, bias and reduce representativeness of the sample.

### Adherence to the preoperative exercise program

The adherence to exercise was higher for the face-to-face sessions (93%) than for the home exercises (64%). The poor adherence from unsupervised home exercises has been previously reported in the literature [29, 31]. A different approach involving a telehealth coach could potentially improve the adherence for the unsupervised exercises. Although this would have to be further studied in this population. In addition, future studies should investigate if the number of exercise sessions (face-to-face or home exercise) is associated with improved postoperative outcomes.

### Acceptability

Based on our semi-structured questionnaire, all participants were either satisfied (33%) or extremely satisfied (67%) with the preoperative exercise program, with none of the patients reporting any negative feedback. This shows that the preoperative exercise protocol was acceptable to patients undergoing pelvic exenteration and CRS & HIPEC, and therefore, a full-scale trial is warranted.

### Secondary outcomes

This pilot trial assessed a wide range of important secondary outcomes that would facilitate the sample size calculation of a full-scale trial. None of the investigated secondary outcomes (e.g. postoperative complications, length of hospital stay, functional capacity, physical activity, quadriceps strength and health-related quality of life) demonstrated a significant difference between the intervention and control group. On this note, this pilot trial was not statistically powered and developed to assess differences between the intervention and control arms; therefore, any perceived trend cannot be interpreted as an indication of an effect and conclusions should not be drawn from the selected secondary outcomes. There is a strong need of a larger sample size to address the secondary outcomes highlighted above. Furthermore, the evaluation of a cost effectiveness analysis should be also explored.

### Other recommendations for future studies

Future full-scale trials investigating the effectiveness of a preoperative exercise on postoperative outcomes in patients undergoing cancer surgery should consider health-related quality of life as one of the outcomes, including physical functioning, role-physical, bodily pain, general health, vitality, social functioning, role-emotional, mental and physical domains [4, 32]. Comparing these outcomes, in addition to others such as the distance walked in the 6-min walking test, with normative values would help with the understanding of how disabled patients were at baseline.

### Limitations

This pilot trial presented some limitations. As the supervised exercise training was conducted once a week for 2 to 6 weeks, some patients received only two face-to-face exercise sessions. The association between volume of exercise and outcomes may need to be further investigated in larger trials, as it is possible that larger amount of exercise sessions could provide further benefits to patients. There are some previous research indicating a positive effect of short-term (1 week) exercise programs in reducing postoperative complications [33, 34]. Another limitation of the current study is that this pilot was conducted in a single centre and, therefore, potential issues related to generalisability, processes and recruitment, were missed. Lastly, the face-to-face intervention was delivered in the local hospital, which excluded many patients from remote regions. Future trials should focus on delivering the face-to-face exercise intervention on local communities or with the use of mobile applications to capture patients that leave far away from main centres. {Steffens, 2020 #76}

## Conclusions

A pilot randomized controlled trial was successfully conducted to investigate the feasibility and acceptability of a preoperative exercise program in patients undergoing pelvic exenteration and CRS & HIPEC. The preoperative program is shown to be feasible and acceptable, suggesting that a full-scale randomized controlled trial should be conducted to evaluate the effectiveness of a preoperative exercise program on the postoperative complication rate in this select group of patients.

## Supplementary Information

**Additional file 1: Supplementary Table 1.** Estimates of physical assessment and self-reported outcome.

## Data Availability

The datasets used and/or analysed during the current study are available from the corresponding author on reasonable request.

## Abbreviations

CRS & HIPEC: Cytoreductive Surgery & Hyperthermic Intraperitoneal Chemotherapy
OR: Odds ratio
CI: Confidence intervals
CPET: Cardiopulmonary exercise
DASS: Depression, Anxiety, Stress Scale
SOuRCe: Surgical Outcomes Research Centre
REDCap: Research Electronic Data Capture
IQR: Interquartile range
CONSORT: Consolidated Standards of Reporting Trials
