# Supplementary material for: Feasibility and acceptability of a preoperative exercise program for patients undergoing major cancer surgery: results from a pilot randomized controlled trial
Source: Pilot Feasibility Stud. 2021 Jan 13;7:27. doi: 10.1186/s40814-021-00765-8 (PMC7805142; doi:10.1186/s40814-021-00765-8)
Supplement: Supplementary file 1 — Additional file 1: Supplementary Table 1. Estimates of physical assessment and self-reported outcome. [file 40814_2021_765_MOESM1_ESM.docx]

| **Supplementary Table 1.** Estimates of physical assessment and self-reported outcomes | | | | | | | | | |
| --- | --- | --- | --- | --- | --- | --- | --- | --- | --- |
| **Variables** | **Baseline** | | **Week before surgery** | | **10 days post-operative** | | **Pre-discharge from hospital** | |  |
|  | **Intervention Group** | **Control Group** | **Intervention Group** | **Control Group** | **Intervention Group** | **Control Group** | **Intervention Group** | **Control Group** | |
| Isometric Quadriceps Strength | 7.5  (4.0 to 13.9) | 9.2  (5.4 to 19.6) | 13.1  (6.4 to 22.5) | 11.2  (4.5 to 19.1) | 2.9  (1.6 to 2.9) | 6.1  (3.2 to 16.7) | 14.6  (2.2 to 32.0) | 5.0  (3.0 to 26.0) | |
| Five times sit to stand | 11.4  (7.6 to 13.1) | 9.8  (7.1 to 12.2) | 11.4  (7.6 to 12.4) | 9.8  (6.5 to 11.4) | 16.5  (3.4 to 21.4) | 15.3  (9.6 to 19.5) | 15.0  (11.3 to 23.0) | 11.0  (7.2 to 14.4) | |
| Six-minutes walking distance (m) | 490.0  (370.0 to 585.0) | 525.0  (459.0 to 585.0) | 460.0  (440.0 to 604.0) | 510.0  (473.5 to 613.5) | 180.0  (60.0 to 267.0) | 149.0  (29.0 to 354.0) | 300.0  (150.0 to 369.5) | 410.0  (240.0 to 460.0) | |
| Pain (VAS) | 4.0  (2.0 to 6.0) | 1.0  (0.0 to 4.0) | 2.0 (2.0 to 5.0) | 2.0 (0.0 to 3.0) | 7.0 (6.0 to 9.0) | 4.5 (3.0 to 6.3) | 4.0 (3.0 to 5.0) | 2.0 (1.0 to 4.3) | |
| Health-related quality of life | | | | | | | | | |
| *Physical component score* | 44.1  (39.3 to 47.8) | 49.6  (46.4 to 52.5) | 46.4  (38.1 to 51.4) | 51.2  (43.7 to 53.2) | 30.7  (18.4 to 38.7) | 29.0  (26.3 to 36.7) | 34.9  (26.1 to 38.7) | 34.6  (32.9 to 44.9) | |
| *Mental component score* | 46.0  (27.6 to 50.6) | 44.3  (37.3 to 56.8) | 44.7  (40.1 to 50.5) | 47.9  (38.2 to 56.9) | 44.8  (34.0 to 46.9) | 35.8  (28.7 to 48.0) | 42.4  (36.3 to 50.3) | 44.0  (33.6 to 50.7) | |
| Data presented as median (interquartile range) | | | | | | | | | |
